# Imaging Infection and Inflammation

**DOI:** 10.1155/2015/615150

**Published:** 2015-03-24

**Authors:** Alberto Signore, Andor W. J. M. Glaudemans, Filippo Galli, François Rouzet

**Affiliations:** ^1^Nuclear Medicine Unit, Department of Medical-Surgical Sciences and Translational Medicine, Faculty of Medicine and Psychology, Ospedale S. Andrea, “Sapienza” University, Via di Grottarossa 1035, 00189 Roma, Italy; ^2^Department of Nuclear Medicine and Molecular Imaging, University Medical Center Groningen University of Groningen, Hanzeplein 1, 9700 RB Groningen, Netherlands; ^3^Nuclear Medicine Department, Bichat-Claude Bernard Hospital and Paris Diderot University, 46 Rue Henri Huchard, 75018 Paris, France

Infectious and inflammatory diseases are a heterogeneous class of diseases encompassing many different clinical conditions and may be classified into infection, acute inflammation, and chronic inflammation. These diseases can affect multiple organs (systemic) or can be localized in one specific organ and are often relapsing and invalidating and may require life-long treatment [1].

Clinicians often struggle with questions in patients dealing with presumed or established infectious and inflammatory disorders. Often they are not well-recognized and when recognized many questions remain: Is there an infectious focus or an inflammatory sign? What are the size and the extent of the lesion? Is this the only lesion in the body or are there infectious metastases? When can the treatment be stopped or should it be switched or continued?

It is very important for the clinician to distinguish between an infection and sterile inflammation. If possible, it may lead to major changes in the treatment of the patients. The recent progress in our understanding and knowledge of the pathophysiology of many infectious and inflammatory diseases has led to the development of several new and specific radiopharmaceuticals in order to meet clinical demand [2–4]. We also learned that, with some radiopharmaceuticals, the lack of specificity can be overcome by applying a particular imaging acquisition protocol (as it is for white-blood-cell scan in prosthetic joint infections) [5] or by using combination of two radiopharmaceuticals (as it is for imaging diabetic foot infection in patients with Charcot joint) [6].

Nowadays, the goal is to identify the type of the disease and the cells and mediators that are involved in the process [7–10].

Diagnosis and characterization of the infectious or inflammatory processes are most often performed with conventional radiology and biochemical tests. Radiological techniques show anatomical changes. These changes, however, usually occur in a late and chronic stage of the disease, thereby leading to a delayed diagnosis and therapy. Nuclear medicine imaging techniques detect biological and biochemical changes in the earliest phase of the diseases, thereby allowing the clinician not only to promptly identify the infective or inflammatory focus but also to establish the best therapeutic approach for patients. The recent availability of many innovative molecules, such as monoclonal antibodies and analogues of growth and signaling factors, offers to nuclear medicine physicians a wide spectrum of promising radiopharmaceuticals as markers for different pathological events.

The development of hybrid camera techniques, such as PET/CT and SPECT/CT, led to a new area of imaging, in which the best of both anatomical and pathophysiological techniques are combined, leading to higher specificities and diagnostic accuracies [11]. The very recent development of combined PET/MRI camera systems may bring this diagnostic accuracy to an even higher level, especially in inflammatory cases, since MRI offers better soft tissue contrast information than CT [12].

This special issue consists of both invited review articles and original research papers from experts from all over the world in the booming field of infectious and inflammatory diseases. The focus of this special issue is to highlight existing imaging techniques and their current position in specific infectious and inflammatory diseases, to share new possibilities in new hybrid camera systems, to identify common patterns for nuclear medicine and radiological diagnosis of infectious and inflammatory diseases, to present new cellular and animal models for preclinical research, and to share the development and testing of novel radiopharmaceuticals in the field.

*Infection and Inflammation of the Cardiovascular System.* Diagnosing infections and inflammation of the cardiovascular system is a rapidly growing area in which nuclear medicine techniques play an important role. Several papers on this topic are published in this special issue dealing with vasculitis, vascular prosthetic graft infection, atherosclerosis, and endocarditis.

H. Balink et al. present an overview of the use of ^18^F-FDG-PET/CT in large vessel vasculitis. They claim that there is a substantial under diagnosis of large vessel vasculitis and that ^18^F-FDG-PET/CT may shed a new light on the classification of giant cell arteritis and Takayasu arteritis, thereby strengthening the idea that both diseases are more likely to be different expressions of a common histopathological entity.

C. Puppo et al. provide a systematic review including 19 original articles with a total of 442 patients with giant cell arteritis. They describe all the different qualitative, semiquantitative, and combined methods that have been proposed for assessing the presence and grading of the severity of the vascular inflammation. Qualitative methods are less sensitive but more specific than semiquantitative methods. Among the semiquantitative approaches, the aortic-to-blood pool uptake ratio of the aortic arch seems to be the most accurate method.

K. J. Lensen et al. evaluated several qualitative/visual analysis methods in this setting and looked at the best interobserver agreement between six nuclear medicine physicians of both academic and peripheral/general hospitals. They found the highest interobserver agreement when vascular wall ^18^F-FDG uptake higher than liver uptake was used as a diagnostic criterion. Agreement was also good without predefined methods, the so-called first impression. They highlighted that standardization of image assessment promotes interobserver agreement.

The paper by B. R. Saleem et al. deals with the same problem of which interpretation criterion should be used but this time in vascular prosthetic graft infections (VPGI). No clear guidelines exist on this area and the interpretation by a visual grading scale is considered to be too subjective. Based on literature they suggest that a linear, diffuse, and homogeneous uptake should not be regarded as an infection, whereas focal or heterogeneous uptake with a projection over the vessel on CT is highly suggestive of infection.

To the opinion of the guest editors, ^18^F-FDG-PET/CT should routinely be used in the imaging of vasculitis and in vascular graft infection, both for diagnosis and for therapy evaluation. Of course it is of invaluable importance that we all follow the same procedures in acquisition protocols and interpretation criteria to make comparison between literature results possible and to start multicenter prospective trials to finally make this technique a new standard in diagnostic and therapy evaluating flow charts.

R. S. Ripa and A. Kjaer share with us the initial experiences with simultaneous PET/MRI for atherosclerosis imaging. They give a short summary of current relevant clinical applications of PET and MRI in the setting of atherosclerosis and discuss future potential vascular applications exploiting the unique combination of PET and MRI.

M. Musso and N. Petrosillo performed a review of the literature to assess the available evidence on the role of nuclear medicine techniques in the diagnosis of prosthetic valve endocarditis. Both ^18^F-FDG-PET/CT and ^99m^Tc-HMPAO labelled white blood cells with SPECT/CT have demonstrated to be effective imaging methods that maybe can be used to increase the sensitivity of the Duke criteria and can identify important complications in the course of prosthetic valve endocarditis, including perivalvular abscesses and metastatic septic embolization. 

*Sarcoidosis.* Sarcoidosis is a granulomatous disease that usually affects multiple organs. The presence of cardiac involvement is associated with adverse events such as heart failure, arrhythmias and sudden cardiac death, and an overall poor outcome. Early diagnosis and treatment is critically important. Therefore, M. Orii et al. provide us an overview of the epidemiology, etiology, histological findings, and clinical features of sarcoidosis. In this paper, they introduce advanced imaging methods including ^18^F-FDG-PET and cardiac MRI as reliable diagnostic modalities for cardiac sarcoidosis. Hepatosplenic involvement of sarcoidosis often shows a benign course but portal hypertension and loss of liver function may occur. Therefore, a correct evaluation of these organs represents an important step in patients with sarcoidosis. The role of conventional imaging, such as CT and MRI in the assessment of hepatosplenic sarcoidosis, is well-established, by revealing organ enlargement, multiple discrete nodules, and lymphadenopathy. C. Tana et al. describe in their review contrast-enhanced ultrasound findings in liver and spleen involvement and share with us evidence from the literature and cases from their own experience. 

*Preclinical Research.* Preclinical research is of invaluable importance to continue look for new radiopharmaceuticals and better use of existing radiopharmaceuticals to improve the diagnostic possibilities. J. C. E. Odekerken et al. focused on the longitudinal assessment of a nonimplant related osteomyelitis by introducing an intramedullary tibial infection with* S. aureus* in NZW rabbits. The development of the infection was assessed by microPET at consecutive time points using ^18^F-FDG as an infection tracer. Indeed, the intramedullary contamination of the rabbit tibia resulted in osteomyelitis, so osteomyelitis in the rabbit can be induced without the use of an implant or sclerosing agent as was used in earlier studies. From all parameters used, the diagnostic value of ^18^F-FDG micro PET was the most versatile to assess the presence of an orthopaedic infection in this animal model.

D. M. Chandrupatla et al. made modifications in a rat model of rheumatoid arthritis (RA) to widen the therapeutic window for PET-guided evaluation of novel anti-RA agents. They induced arthritis in the right knee of Wistar rats and gave, after immunization, one or more intra-articular methylated bovine serum albumin injections over time in the right knee. Besides many other analyses, they found increased ^18^F-FDG and ^11^C-PK11195 accumulation in arthritic knees as compared to contralateral normal knees, which was confirmed in* ex vivo* tissue distribution studies. This model will offer excellent opportunities for repeated PET studies to monitor progression of disease and efficacy of novel therapeutic agents for RA in the same animal.

Antimicrobial peptides are a heterogeneous class of compounds found in a variety of organisms including humans. The ability of these peptides to accumulate at sites of infection combined with the minimal host's cytotoxicity makes these peptides potential radiopharmaceuticals to allow noninvasive whole-body examination for detection of occult infection. An overview of all the existing peptides and the strategy, design, and utilization of these peptides is given by T. Ebenhan et al.

B. Mokaleng et al. report TBIA101, an antimicrobial peptide derivate that was conjugated to DOTA and radiolabelled with ^68^Ga for an* in vitro* and* in vivo* infection imaging using* Escherischia coli*-bearing mice by targeting bacterial lipopolysaccharides with PET/CT. ^68^Ga-DOTA-TBIA101-PET detected* E. coli*-infected muscle tissue > noninfected thighs > forearm muscles > background in the same animal. Normalization of the infected thigh muscle to reference tissue showed a ratio of 3.0 ± 0.8 compared to a ratio of 2.3 ± 0.6 in the identical healthy tissue. These findings warrant further preclinical imaging studies of greater depth. 

*Tailored Radiopharmaceuticals.* Nuclear medicine techniques may play a crucial role by providing objective biomarkers for personalized medicine. These techniques offer the unique possibility to target cells and molecules that are involved for histological characterization, selection of patients for targeted therapy, therapy response prediction, and therapy response follow-up. Research in this field must be encouraged and supported.

The paper from F. Galli et al. is a nice example of this important concept. Recently, the use of anti-tumor necrosis factor alpha (anti-TNF*α*) agents has been introduced for therapy of chronic and refractory sarcoidosis with controversial results. Therefore, ^99m^Tc-labelled infliximab was used to study the expression of TNF*α* in sarcoid lesions and to evaluate its role as a predictive marker of response to therapy. The results were, however, slightly disappointing. A low correlation was found between target-to-background ratios and BAL results in patients that were despite positive at ^18^F-FDG-PET. The authors concluded that in this study 10 patients had a low presence of TNF*α*. The concept that this anti-TNF*α* scintigraphy is a good tool for the selection of patients to be treated with anti-TNF*α* drugs is an important one since our techniques can reveal if this expensive therapy will be successful in the individual patient. So maybe the 10 patients scanned should not be treated with anti-TNF*α* drugs; however, to conclude this further investigation is warranted.

*Alberto Signore* *Andor W. J. M. Glaudemans* *Filippo Galli* *François Rouzet*
